# Second primary acute lymphoblastic leukemia in adults: a SEER analysis of incidence and outcomes

**DOI:** 10.1002/cam4.1266

**Published:** 2017-12-28

**Authors:** Abhisek Swaika, Ryan D. Frank, Dongyun Yang, Laura E. Finn, Liuyan Jiang, Pooja Advani, Asher A. Chanan‐Khan, Sikander Ailawadhi, James M. Foran

**Affiliations:** ^1^ Division of Hematology & Medical Oncology Mayo Clinic Jacksonville Florida; ^2^ Division of Biomedical Statistics and Informatics Mayo Clinic Rochester Minnesota; ^3^ Department of Preventive Medicine Norris Comprehensive Cancer Center University of Southern California Los Angeles California; ^4^ Division of Hematopathology Department of Pathology and Laboratory Medicine Mayo Clinic Jacksonville Florida; ^5^ Mayo Clinic Cancer Center Jacksonville Florida

**Keywords:** Second primary acute lymphoblastic leukemia, second primary ALL, SEER analysis, SIRs, standardized incidence ratios

## Abstract

We conducted a surveillance epidemiology and end results (SEER)‐based analysis to describe the incidence and characteristics of second primary acute lymphoblastic leukemia (sALL) among adults (≥18 years) with a history of primary malignancies (1M). Standardized incidence ratios (SIRs) of sALL cases were calculated by site and 1M stage. We also evaluated the differences in 5‐year sALL survival by age, site, and extent of 1M, latency of sALL after 1M, and evidence of underlying racial/ethnic disparity. We identified 10,956 patients with de‐novo/primary acute lymphoblastic leukemia (1ALL) and 772 with sALL. Women (49.1% vs. 42.9%), white patients (72.0% vs. 59.5%), older patients (58.8% vs. 25.2%; age ≥65 years), and patients diagnosed between 2003 and 2012 (66.8% vs. 53.9%) had a higher proportion of sALL compared with 1ALL. There was a significantly inferior median 5‐year survival for sALL patients compared to 1ALL (6 vs. 15 months; HR 1.20, 95% CI 1.10–1.31, *P* < 0.001). The median latency period was 60.0 months; the most common 1M among sALL patients were breast (17.9%) and prostate (17.4%). Patients with any 1M were at increased risk of developing sALL (SIR 1.76, 95% CI 1.58–1.95, *P* < 0.001). Hematological‐1M sites had significantly higher SIRs (hematological‐SIR 7.35; solid‐SIR 1.33; *P* < 0.001). We observed a significant increase in sALL incidence after a 1M and a significantly worse 5‐year survival with different demographic characteristics from 1ALL. There is a need to define appropriate screening methods for patients surviving their primary cancer.

## Introduction

Acute lymphoblastic leukemia (ALL) is the most common form of childhood leukemia worldwide and in the United States; 40% of all the newly diagnosed ALL cases occur in adults older than 20 years of age 1. Approximately 6250 new cases of ALL were diagnosed in 2015, and of which, approximately one‐quarter were older than 45 years of age, whereas patients older than 65 years comprised more than 10% of new cases (http://seer.cancer.gov/statfacts/html/alyl.html) 1.

Compared to the general population, cancer survivors have a 14% increased risk of developing another malignancy 2. With major advancements in the treatment of various hematological and solid organ malignancies, there has been an increase in patient overall survival. This resultant and encouraging increase in life expectancy has also fueled a renewed concern about long‐term comorbidities, including the risk of secondary malignancies 3.

Second primary ALL (sALL) in adults that arises after a previous diagnosis of a primary malignancy is a rare and an underrecognized entity. It is thought to represent 2–10% of all cases of diagnosed ALL, according to previous small‐population and single‐institutional studies 4, 5, 6, 7, 8, 9. Prior exposure to chemotherapy or radiotherapy has been postulated as a possible independent risk factor for the development of sALL with the occurrence of adverse cytogenetic abnormalities, and therefore there is possible association with poor prognosis, though conflicting reports exists 5, 10, 11, 12. Immunodeficiency in the form of either malignancy, antineoplastic treatment, auto‐immune disorders, and immunomodulation (e.g., in patients treated with lenalidomide) have also been described as risk factors for the development of lymphoblastic leukemia/lymphoma 5, 6, 10, 12, 13, 14.

Therapy‐related acute myeloid leukemia is now recognized as a defined clinical disease entity in the World Health Organization (WHO) classification of myeloid neoplasms and acute leukemia; however, no consensus or WHO classification exists for sALL 15.

Observations in our own clinical practice, wherein previously treated malignancies (mostly localized breast cancer) in newly diagnosed ALL and the paucity of substantial data on the incidence of sALL and outcomes of sALL patients compelled us to conduct this comprehensive surveillance epidemiology and end results (SEER)‐based analysis. We specifically evaluated differences in sALL incidence and survival on the basis of age, sex, year of diagnosis, and SEER registry site, and the extent (local/regional vs. advanced stage) of primary malignancy (1M). We also looked for evidence of any underlying racial/ethnic disparities. Finally we explored whether an earlier versus late occurrence of sALL had any impact on overall survival.

## Methods

### Patients/study population

ICD‐O3 diagnosis codes 9811, 9812, 9814–9818, 9826, 9827, and 9835–9837 were used to identify adult patients (≥ 18 years) with ALL using the case listing tool in SEER*Stat version 8.2.1. Eighteen SEER registries from the years 1973 to 2012 were used. In total, 11,728 patients with ALL were identified and were classified as having primary ALL or sALL using the primary malignancy indicator variable in SEER*Stat. Every tumor in SEER is considered a new primary based on SEER multiple primary rules 16. Latency was defined as the interval time between 1M and sALL. We excluded sALL cases identified by biopsy or death certificate only and cases with a diagnosis of In situ 1M. Patients with 1M diagnosis within 2 months prior to sALL diagnosis were also excluded to confirm a metachronous and not a synchronous diagnosis of 1M and sALL 17. Due to the similarities with ALL, patients with 1M acute lymphocytic leukemia or other acute leukemia were classified as de novo (3 patients) for the survival analyses and excluded from the multiple primary standardized incidence ratios (SIRs) analyses. Demographic information on race/ethnicity (using the SEER*Stat variables Race_recode_W_B_AI_API and OriginrecodeNHIAHispanicNonHis), sex, age, month, and year of diagnosis, vital status, and duration of follow‐up were collected for all patients. The SEER historic A variable was used for staging information of the 1M. Hematological malignancies were defined as distant. 1M sites were classified as solid (colorectal, lung, melanoma of the skin, breast, corpus and uterus, ovary, prostate, bladder kidney, brain, or thyroid) or hematological (lymphoma, myeloma, or other leukemia). Other types of leukemia included chronic lymphocytic leukemia, other nonacute lymphocytic leukemia, acute myeloid leukemia, acute monocytic leukemia, chronic myeloid leukemia, other myeloid/monocytic leukemia, acute lymphocytic leukemia, acute undifferentiated leukemia, acute phenotypic leukemia, mixed phenotype acute leukemia, and acute panmyelosis with myelofibrosis. Lymphoma included Hodgkin – nodal, Hodgkin – extra nodal, NHL – nodal, and NHL – extra nodal. This project was exempt from institutional review board review.

### Statistical analysis

Pearson *χ* ^2^ tests were used to compare the order of ALL diagnoses across levels of the summarized variables. For those with sALL, the latency period was summarized by primary malignancy site using medians and ranges. Duration of follow‐up (in months) was defined as the time from ALL diagnosis to death, censor, or loss to follow‐up.

Five‐year survival was used as the primary endpoint; observations with five or more years of follow‐up were censored at 5 years. Those with unknown survival time were not included in the analysis (5 sALL and 82 1ALL). Kaplan–Meier curves were used to examine the risk of death by order of ALL. A log‐rank test was used to formally compare the differences. Associations between sALL and survival were performed using Cox proportional hazards regression. The following covariates were examined: 1M versus sALL, year, race, sex, and SEER registry. Each covariate was fit univariately in separate Cox regression models. One overall multivariate cox model including all covariates was used to assess independent effects. Each model was stratified by age of diagnosis. The effect of latency time was assessed in a separate set of Cox models.

Multiple primary SIRs and corresponding 95% confidence intervals were used to examine the incidence of sALL by primary malignancy site and stage. SIRs are calculated by dividing the number of observed cases of sALL by the number of population‐based expected counts. The number of expected were calculated by apportioning each observation's follow‐up into age and calendar period categories. Sex, race, and SEER registry site‐specific incidence rates were also used to account for the differences associated with these variables. An SIR > 1.0 can be interpreted as having more observed than expected events. The absolute excess risk was calculated as the difference between the number of observed and the number predicted per 100,000 person‐years of follow‐up. The absolute excess risk > 0.0 can be interpreted as the number of additional observed versus expected cases of ALL per 100,000 person‐years of follow‐up. The number of observations, predicted events, and observed events for the SIRs were obtained using SEER*Stat 17. The SIRs were then calculated using Poisson regression analysis, with the log transformed expected event rate modeled as the offset term. SIRs were calculated by site and stage of 1M. Tests of heterogeneity were performed to detect differences between solid and hematological sites. The staging variables were inconsistent on the earlier years of the SEER registries. Consequently, only 13 registries from the more recent years of 1992–2012 were used for the SIRs analyses.

The survival and multiple primary SIR analyses were repeated with the 1M leukemia patients (including acute lymphocytic leukemia, acute undifferentiated leukemia, acute phenotypic leukemia, mixed phenotype acute leukemia, and acute panmyelosis with myelofibrosis) excluded in sensitivity analyses. *P* < 0.05 was considered significant. All analyses were performed using SAS software version 9.3 (SAS Institute, Inc., Cary, NC, USA).

## Results

A total of 10,956 adult patients with 1ALL and 772 with sALL were identified. Table 1 summarizes the patient demographics. Statistically significant differences were noted between patients with sALL and 1ALL with respect to female sex (49.1% vs. 42.9%), race (72.0% vs. 59.5% white), age at diagnosis (58.8% vs. 25.2% ≥65 years), and year of diagnosis (66.8% vs. 53.9% 2003–2012).

**Table 1:** Demographics of 1ALL versus sALL

Characteristic	ALL de novo (*N* = 10,956)	ALL secondary (*N* = 772)	Total (*N* = 11,728)	*P*‐value^a^
Gender				
Female	4695 (42.9%)	379 (49.1%)	5074 (43.3%)	0.001
Male	6261 (57.1%)	393 (50.9%)	6654 (56.7%)	
Race				
White	6519 (59.5%)	556 (72.0%)	7075 (60.3%)	<0.001
Black	861 (7.9%)	60 (7.8%)	921 (7.9%)	
Asian	817 (7.5%)	54 (7.0%)	871 (7.4%)	
Hispanic	2598 (23.7%)	97 (12.6%)	2695 (23.0%)	
Native American	111 (1.0%)	4 (0.5%)	115 (1.0%)	
Unknown	50 (0.5%)	1 (0.1%)	51 (0.4%)	
Age at diagnosis				
18–44	5030 (45.9%)	74 (9.6%)	5104 (43.5%)	<0.001
45–54	1614 (14.7%)	82 (10.6%)	1696 (14.5%)	
55–64	1549 (14.1%)	162 (21.0%)	1711 (14.6%)	
65–74	1303 (11.9%)	201 (26.0%)	1504 (12.8%)	
75+	1460 (13.3%)	253 (32.8%)	1713 (14.6%)	
Year of ALL diagnosis				
1973–1982	758 (6.9%)	19 (2.5%)	777 (6.6%)	<0.001
1983–1992	1308 (11.9%)	61 (7.9%)	1369 (11.7%)	
1993–2002	2980 (27.2%)	176 (22.8%)	3156 (26.9%)	
2003–2012	5910 (53.9%)	516 (66.8%)	6426 (54.8%)	

1ALL, primary acute lymphoblastic leukemia; ALL, acute lymphoblastic leukemia; sALL, second primary acute lymphoblastic leukemia.

Person Chi‐square *P*‐value.

The median latency period between 1M and sALL diagnosis was 60 months (Table 2, range: 2–423 months). Patients with a previous history of lymphoma and/or previous primary leukemia and lung malignancies had the shortest latency period, whereas patients with previous ovarian (92 months) or thyroid cancer (86 months) had the longest interval, possibly reflecting the natural history of the underlying 1M.

**Table 2:** Latency period by primary site among patients with sALL and a known primary site according to the frequency of 1M

Primary site	*N* (%)	Mean (SD)	Median (Q1–Q3)	Range
All sites	772	72.8 (61.6)	60.0 (24.0–101.0)	2–423
Breast	138 (17.9%)	85.9 (72.4)	67.0 (31.0–114.0)	4–423
Prostate	134 (17.4%)	73.1 (53.9)	66.5 (29.0–94.0)	2–272
Other	112 (14.5%)	69.4 (62.5)	55.5 (19.0–94.0)	2–308
Other Leukemia^a^	87 (11.3%)	46.0 (46.5)	28.0 (13.0–65.0)	2–253
Lymphoma^b^	83 (10.8%)	64.0 (63.2)	42.0 (13.0–104.0)	2–271
Colorectal	53 (6.9%)	82.5 (70.3)	68.0 (33.0–103.0)	3–282
Lung	33 (4.3%)	45.9 (47.3)	36.0 (15.0–53.0)	2–209
Thyroid	29 (3.8%)	85.0 (36.3)	86.0 (54.0–102.0)	22–165
Bladder	24 (3.1%)	98.1 (67.8)	81.0 (53.5–143.0)	6–287
Myeloma	21 (2.7%)	76.5 (41.7)	70.0 (61.0–82.0)	19–214
Melanoma of the skin	20 (2.6%)	77.9 (57.1)	69.5 (39.0–96.5)	2–224
Kidney	20 (2.6%)	84.9 (51.0)	67.5 (50.5–129.0)	3–169
Ovary	16 (2.1%)	103.9 (74.1)	92.0 (42.0–150.5)	15–285
Brain	2 (0.3%)	114.5 (113.8)	114.5 (34.0–195.0)	34–195

1M, primary malignancies; *N*, number; Q1, first quartile; Q3, third quartile; sALL, second primary acute lymphoblastic leukemia; SD, standard deviation.

a Other types of leukemia included chronic lymphocytic leukemia, other nonacute lymphocytic leukemia, acute myeloid leukemia, acute monocytic leukemia, chronic myeloid leukemia, other myeloid/monocytic leukemia, acute lymphocytic leukemia, acute undifferentiated leukemia, acute phenotypic leukemia, mixed phenotype acute leukemia, and acute panmyelosis with myelofibrosis.

b Lymphoma included Hodgkin – nodal, Hodgkin – extra nodal, NHL – nodal, and NHL – extra nodal.

**Figure 1:**
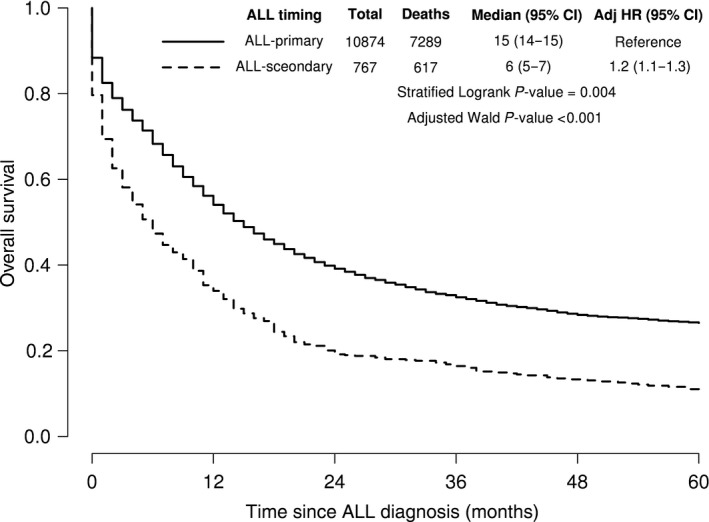
Kaplan–Meier curves of 5‐year survival among ALL patients by timing of ALL. HR is stratified on age of diagnosis and adjusted for year of diagnosis, gender, race, and SEER site.

The median follow‐up for all 11,641 patients with complete follow‐up was 11 months. A total of 7906 deaths were observed within 5 years. sALL patients had significantly inferior median survival compared to 1ALL (6 months vs. 15 months; *P*‐value = 0.004) (Fig. 1). sALL was significantly associated with inferior 5‐year survival after adjustment for year of diagnosis, race, sex, site, and stratifying by age at diagnosis (Table 3; HR 1.20 95% CI 1.10–1.30; overall *P* < 0.001). The same association was observed when all 1M Leukemias were removed (HR = 1.20, 95% CI 1.10–1.31, *P* < 0.001) (data not shown). Latency period had no impact on survival outcomes (Table 4).

**Table 3:** Associations with 5‐year survival using univariate and multivariate cox regression stratified by age of ALL

			Univariate		Multivariate^b^	
Characteristic	Total (*N* = 11,641)	Deaths (*N* = 7906)	Hazards ratio (95% CI)	Wald *P*‐value^a^	Hazards ratio (95% CI)	Wald *P*‐value^a^
ALL timing						
ALL‐primary	10,874	7289 (67.0%)	1.00 (ref)	0.008	1.00 (ref)	<0.001
ALL‐secondary	767	617 (80.4%)	1.12 (1.03, 1.22)		1.20 (1.10, 1.30)	
Year of ALL diagnosis						
1973–1982	768	671 (87.4%)	1.00 (ref)	<0.001	1.00 (ref)	<0.001
1983–1992	1358	1113 (82.0%)	0.88 (0.80, 0.97)		0.87 (0.79, 0.96)	
1993–2002	3126	2344 (75.0%)	0.75 (0.69, 0.82)		0.71 (0.64, 0.77)	
2003–2012	6389	3778 (59.1%)	0.60 (0.55, 0.65)		0.55 (0.50, 0.60)	
Race						
White	7011	4927 (70.3%)	1.00 (ref)	<0.001	1.00 (ref)	<0.001
Black	912	668 (73.2%)	1.31 (1.21, 1.42)		1.41 (1.29, 1.53)	
Asian	867	555 (64.0%)	0.99 (0.91, 1.09)		1.08 (0.98, 1.20)	
Hispanic	2685	1653 (61.6%)	1.10 (1.03, 1.16)		1.18 (1.10, 1.26)	
Native American	115	82 (71.3%)	1.30 (1.05, 1.62)		1.25 (0.98, 1.59)	
Unknown	51	21 (41.2%)	0.66 (0.43, 1.01)		0.74 (0.48, 1.14)	
Gender						
Female	5028	3456 (68.7%)	1.00 (ref)	0.030	1.00 (ref)	0.031
Male	6613	4450 (67.3%)	1.05 (1.00, 1.10)		1.05 (1.00, 1.10)	

ALL, acute lymphoblastic leukemia; No, number age of diagnosis was included as a stratification term in both univariate and multivariate models.

a Overall Type‐3 Wald *P*‐value. Although the confidence intervals for some HRs may contain 1, the *P*‐value tests to see if all levels of the covariate are equal to the referent group, or if one or more levels are significantly different.

b SEER registry also included as an adjustment term in the multivariate model, data not shown.

**Table 4:** Associations with 5‐year survival using univariate and multivariate cox regression on sALL patients with primary site known

			Univariate^a^		Multivariate^a^^,^ b	
Characteristic	Total (*N* = 766)	Deaths (*N* = 617)	Hazards ratio (95% CI)	*P*‐value^c^	Hazards ratio (95% CI)	*P*‐value^a^
Latency period						
<2 years	180	142 (78.9%)	1.00 (ref)	0.592	1.00 (ref)	0.905
2–5 years	198	159 (80.3%)	0.94 (0.74, 1.18)		1.01 (0.80, 1.27)	
5–10 years	248	202 (81.5%)	0.87 (0.70, 1.08)		0.94 (0.75, 1.18)	
10+ years	140	114 (81.4%)	0.97 (0.75, 1.25)		1.01 (0.78, 1.32)	
Year of ALL diagnosis						
1973–1982	19	17 (89.5%)	1.00 (ref)	0.007	1.00 (ref)	0.002
1983–1992	61	59 (96.7%)	1.02 (0.59, 1.76)		1.02 (0.59, 1.77)	
1993–2002	176	160 (90.9%)	1.06 (0.64, 1.75)		1.06 (0.64, 1.78)	
2003–2012	510	381 (74.7%)	0.78 (0.48, 1.27)		0.75 (0.46, 1.25)	
Race						
White	552	454 (82.2%)	1.00 (ref)	0.792	1.00 (ref)	0.521
Black	60	46 (76.7%)	1.06 (0.77, 1.45)		1.12 (0.81, 1.54)	
Asian	53	41 (77.4%)	1.09 (0.79, 1.50)		1.20 (0.86, 1.67)	
Hispanic	97	74 (76.3%)	1.10 (0.85, 1.42)		1.19 (0.91, 1.55)	
Native American	4	2 (50.0%)	0.52 (0.13, 2.09)		0.60 (0.15, 2.47)	
Gender						
Female	377	305 (80.9%)	1.00 (ref)	0.905	1.00 (ref)	0.342
Male	389	312 (80.2%)	0.99 (0.84, 1.16)		1.11 (0.90, 1.37)	

ALL, acute lymphoblastic leukemia; sALL, second primary acute lymphoblastic leukemia.

a Age of diagnosis was included as a stratification term in both univariate and multivariate models.

b Primary malignancy site was included as an adjustment term in multivariate analyses, data not shown.

c Overall Type‐3 Wald *P*‐value. Although the confidence intervals for some HRs may contain 1, the *P*‐value tests to see if all levels of the covariate are equal to the referent group, or if one or more levels are significantly different.

Any prior cancer history increased the risk of developing ALL (Table 5, SIR 1.76, 95% CI 1.58–1.95, *P* < 0.001). This comparison remained significant when 1M Leukemias were removed (SIR 1.51, 95% CI 1.35–1.70) (data not shown). Significantly higher SIRs were observed for breast (1.87), other non‐ALL subtypes of leukemia (14.72), myeloma (6.58), lymphoma (4.45), thyroid (3.85), lung (1.93), brain (5.59), and ovarian (2.65), suggesting that these types of 1M are more likely to be predisposed to development of sALL. There were no significant differences in prostate, colorectal, kidney, corpus and uterus, bladder, or melanoma of the skin. The absolute excess risks for sALL from all sites was 1.01 (0.78–1.27; *P* < 0.001) additional cases per 100,000 person‐year follow‐up.

**Table 5:** Number of observed versus expected sALL cases by site of primary malignancy

Cancer site	Persons	Observed events	Expected events	SIRs^a^ (95% CI)	Absolute excess risk^a^ no./100,000 person‐year (95% CI)	*P*‐value^b^
All sites	2,693,360	341	194.02	1.76 (1.58, 1.95)	1.01 (0.78, 1.27)	<0.001
Breast	437,473	63	33.70	1.87 (1.46, 2.39)	0.94 (0.50, 1.51)	<0.001
Prostate	496,994	58	60.74	0.95 (0.74, 1.24)	−0.08 (−0.45, 0.42)	0.725
Other leukemia^c^^,^ d	59,123	53	3.60	14.72 (11.25, 19.27)	18.64 (13.93, 24.82)	<0.001
Other^e^	457,868	41	21.52	1.91 (1.40, 2.59)	1.10 (0.49, 1.93)	<0.001
Lymphoma^f^	134,350	39	8.76	4.45 (3.25, 6.09)	4.08 (2.66, 6.01)	<0.001
Thyroid	66,182	16	4.16	3.85 (2.36, 6.28)	2.51 (1.20, 4.65)	<0.001
Colorectal	268,187	14	19.80	0.71 (0.42, 1.19)	−0.42 (−0.83, 0.27)	0.195
Lung	280,881	14	7.24	1.93 (1.15, 3.27)	1.29 (0.21, 3.13)	0.014
Myeloma	34,139	10	1.52	6.58 (3.54, 12.23)	7.57 (3.45, 15.24)	<0.001
Brain	37,120	9	1.61	5.59 (2.91, 10.74)	5.87 (2.44, 12.46)	<0.001
Kidney	71,040	7	4.76	1.47 (0.70, 3.08)	0.65 (−0.41, 2.86)	0.308
Corpus and uterus	87,343	5	6.50	0.77 (0.32, 1.85)	−0.26 (−0.77, 0.96)	0.557
Ovary	42,095	5	1.89	2.65 (1.10, 6.36)	1.63 (0.10, 5.32)	0.030
Bladder	109,609	4	9.53	0.42 (0.16, 1.12)	−0.91 (−1.32, 0.19)	0.083
Melanoma of the skin	110,956	3	8.69	0.35 (0.11, 1.07)	−0.74 (−1.00, 0.08)	0.065

sALL, second primary acute lymphoblastic leukemia; SIR, standardized incidence ratio.

a SIRs compares the number of observed to expected sALL cases on the basis of SEER incidence data. All analyses account for the effects of age, sex, race, SEER registry site, and calendar period.

b Type 3 Wald test.

c Primary malignancies of acute lymphocytic leukemia and other acute leukemia were excluded.

d Other types of leukemia included chronic lymphocytic leukemia, other nonacute lymphocytic leukemia, acute myeloid leukemia, acute monocytic leukemia, chronic myeloid leukemia, and other myeloid/monocytic leukemia.

e Any cancer type with fewer than 3 total events were classified as other.

f Lymphoma included Hodgkin – nodal, Hodgkin – extra nodal, NHL – nodal, and NHL – extra nodal.

Patients with distant extent had higher risk of sALL than other extents (Table 6). The SIRs for localized (1.31), regional (1.48), and distant (5.44) patients were significantly different (*P* < 0.001). There was no evidence for significant differences by disease extent for any of the most common 1M including breast, prostate, colorectal, or lung (Table 6). Hematological tumors had significantly higher SIRs than solid tumor 1M (hematological: 7.35, 95% CI 6.05–8.92; solid‐SIR 1.33, 95% CI 1.17–1.51; *P* < 0.001; data not shown). When subset to solid 1M, there was no difference by extent (localized/regional SIR 1.24, distant SIR 1.88, *P* = 0.157). In distant (advanced stage) patients, Hematological 1M sites had higher sALL risk than solid tumors (hematological SIR 7.35, 95% CI 6.05–8.92; solids SIR 1.88, 95% CI 1.11–3.17; *P* < 0.001).

**Table 6:** Number of observed versus expected sALL cases by stage and site of primary malignancy

Cancer site	Persons	Observed events	Expected events	SIRs^a^ (95% CI)	*P*‐value^b^
All sites					
Localized	906,666	98	74.87	1.31 (1.07, 1.60)	<0.001
Regional	522,995	45	30.38	1.48 (1.11, 1.98)	
Localized/regional^c^	389,132	43	45.02	0.96 (0.71, 1.29)	
Distant	597,664	116	21.34	5.44 (4.53, 6.52)	
Breast					
Localized	272,487	40	23.41	1.71 (1.25, 2.33)	0.275
Regional	132,741	21	9.11	2.31 (1.50, 3.54)	
Distant	24,182	0	0.71	–	
Prostate					
Localized/regional^c^	389,132	43	45.02	0.96 (0.71, 1.29)	0.884
Distant	18,790	1	0.90	1.11 (0.16, 7.89)	
Other leukemia^d^^,^ e					
Distant	59,123	53	3.60	14.72 (11.25, 19.27)	–
Other^f^					
Localized	142,708	14	10.28	1.36 (0.81, 2.30)	0.321
Regional	130,558	11	5.99	1.84 (1.02, 3.32)	
Distant	86,425	5	1.62	3.09 (1.28, 7.42)	
Lymphoma^g^^,^ h					
Distant	134,350	39	8.76	4.45 (3.25, 6.09)	
Thyroid					
Localized	39,306	12	2.48	4.84 (2.75, 8.52)	0.144
Regional	22,887	2	1.44	1.39 (0.35, 5.55)	
Distant	2709	1	0.14	7.14 (1.01, 50.71)	
Colorectal					
Localized	109,814	8	10.49	0.76 (0.38, 1.52)	0.926
Regional	99,372	5	7.74	0.65 (0.27, 1.55)	
Distant	48,290	1	1.06	0.94 (0.13, 6.70)	
Lung					
Localized	51,625	8	2.96	2.70 (1.35, 5.40)	0.481
Regional	75,017	3	2.42	1.24 (0.40, 3.84)	
Distant	135,990	3	1.47	2.04 (0.66, 6.33)	

sALL, second primary acute lymphoblastic leukemia; SIR, standardized incidence ratio.

a SIRs compares the number of observed to expected sALL cases on the basis of SEER incidence data. All analyses account for the effects of age, sex, race, SEER registry site, and calendar period.

b Test of heterogeneity across levels of stage. Only performed if 2 or more stages had an observed event.

c Only defined for prostate cancer.

d Primary malignancies of acute lymphocytic leukemia and other acute leukemia were excluded.

e Other types of leukemia included chronic lymphocytic leukemia, other non‐acute lymphocytic leukemia, acute myeloid leukemia, acute monocytic leukemia, chronic myeloid leukemia, and other myeloid/monocytic leukemia.

f Any cancer type with fewer than 3 total events were classified as other.

g All cases of Lymphoma were set to stage = distant.

h Lymphoma included Hodgkin – nodal, Hodgkin – extra nodal, NHL – nodal, and NHL – extra nodal.

## Discussion

We demonstrate that patients with any previous malignancy had an increased incidence of sALL cases (SIR 1.76), which establishes sALL as a distinct entity. As demonstrated, sALL has distinct characteristics compared to de novo ALL. It should be identified by clinicians as a separate diagnosis that should be actively screened, especially as a part of cancer survivorship. Specifically, patients with a previous primary diagnosis of breast, other leukemia, lymphoma, thyroid, myeloma, brain, and ovarian cancers had a significantly higher incidence of developing sALL, as evidenced by higher SIRs for these 1M diagnoses. From all sites of 1M, there was ~ 1 (absolute excess risk 1.01, 95% CI 0.78–1.27) additional sALL case for every 100,000 person‐years of follow‐up. The 1M sites with the highest absolute excess risk per were other leukemia (18.64), myeloma (7.57), and brain (5.87). Interestingly, we observed that patients with a previous primary hematological malignancy had a higher risk for sALL as compared to solid organ neoplasms.

We also established that sALL was associated with a significantly lower median survival as compared to de novo ALL (6–15 months). Importantly, these survival differences remained significant after adjustment for year of diagnosis, race, gender, and SEER registry site. Contrary to our initial supposition, we did not find any evidence for association between latency period of sALL diagnosis and survival, with the observed median latency period being 60 months.

In this study, sALL comprised 6.6% of cases, which was similar to other published data 7, 8. Pagano et al. 4 reported an incidence of 2.3% from 901 cases during the years 1992–1996, whereas Ganzel et al. observed a sALL incidence rate of 9.4% from a single U.S. institution 11. Most of the previously published studies are on small archives or single institution patient population. We do note a recent follow‐up publication from SEER initiated after our study was first reported in abstract form in 2014, and based on a subset of patients who were also included in this larger and more comprehensive long‐term analysis. Importantly that analysis also confirms the results of this larger and unselected cohort that we report, showing a similar incidence and adverse impact on survival 9, 18.

Our results demonstrate significant heterogeneity in risk of sALL by age, sex, race, and histology of 1M diagnosis. We observed a higher percentage of women with sALL then women with 1ALL, which was in contrast to earlier reports demonstrating a male preponderance for sALL except for the analysis by Giri et al. 4, 8, 9. The variability in proportion of sALL based on race was notable. Whites had a higher percentage of sALL than 1ALL, whereas Hispanics had a lower percentage of sALL than 1ALL. While the definition of race has varied over time SEER, post hoc sensitivity analyses subset to ALL diagnosed after 1993, when race was more consistently defined, revealed the same results (Table S1). This difference could be due to a multitude of causes, including but not limited to interactions between genetic‐environmental factors, regular follow‐up/surveillance, level of awareness, or even access to healthcare post treatment of 1M. Socioeconomic status and access to healthcare has been ascertained as one of the primary reasons for different outcomes in cancer care including survival 19. Geocoding (i.e., use of address/postal code at diagnosis) could be used as a surrogate for socioeconomic status in future studies, but unfortunately this information was not included in the SEER database and was not used in this analysis.

It was not surprising that breast (17.9%) and prostate (17.4%) 1M had the most cases of sALL, as these are the most common cancers in men and women, respectively 20, 21. Relatively few cases of sALL were observed following lung cancer, the second most common malignancy in both men and women 22. Given the poor prognosis of lung cancer however, this was not surprising. The highest risk of developing sALL was when the primary malignancy was hematological in nature. This could potentially highlight the similarity in disease biology in terms of leukemic plasticity or similar cytogenetic abnormalities. Concerns on treatment modalities have previously showed a concordance with increased risks of secondary malignancies, including non‐Hodgkin lymphomas / multiple myeloma, and could significantly contribute to the observed higher incidence of sALL 14.

A major strength of our current analysis was the study sample coming from a large population‐based registry over many years with extended durations of follow‐up. To our knowledge, this is the largest and most current population‐based study to evaluate the incidence of sALL in adult patients. Other strengths inherent to the SEER database include standardized confirmed diagnostic criteria and broad long‐term representation of the U.S. population.

There are some limitations to using SEER data that should be acknowledged. Cytogenetics and genetic details are not captured in SEER data. Consequently, we were unable to describe the possible differences in disease phenotypes, including presence of specific sALL cytogenetic or genetic lesions. Prior reports on secondary acute myeloid leukemia have observed differences, and there are likely the same differences in sALL. Additionally, the SEER database does not have detailed information regarding long‐term treatment and treatment responses prior to therapy of the primary or secondary malignancy. We were unable to determine whether the higher incidence of sALL represented an effect of treatment for 1M, an immunosuppressive effect of prior therapy, or an inherent increased predisposition to developing secondary malignancies. Furthermore, the definition of race varied over time in SEER databases before 1992 and after 1992, with the races/ethnicity Hispanic and Asian having lower capture rates in the earlier years than the later years. However, results from post hoc sensitivity analyses subset to the years 1993 to 2012 (Tables S1–S2 and Fig. S1) were consistent with the primary analysis, so any impact the inconsistencies had on the primary analysis were minimal.

Future, coordinated, prospective studies are needed to help determine whether those with sALL are at higher risk of developing adverse cytogenetic lesions, or whether they may benefit from alternative treatment strategies, including allogenic transplantation. The proposed study would provide more detailed insights into the clinical and genetic risk factors for developing sALL, which may help in future screening and prevention strategies for this uncommon and adverse disease entity.

## Disclosures

Ailawadhi reports personal fees from Takeda Oncology, personal fees from Novartis Pharmaceuticals, personal fees from Amgen Pharmaceuticals, personal fees from Pharmacyclics, outside the submitted work. The other authors have nothing to disclose. SA receives consultant fees from Takeda Oncology.

## Conflict of Interest

The authors have no conflict of interests to disclose.

## Supporting information


**Figure S1.** Kaplan–Meier curves of 5‐year survival among ALL patients diagnosed between 1993 and 2012 by timing of ALL. HR is stratified on age of diagnosis and adjusted for year of diagnosis, gender, race, and SEER site.

**Table S1.** Demographics of 1ALL versus sALL in patients diagnoses between 1993 and 2012.
**Table S2.** Associations with 5‐year survival using univariate and multivariate cox regression stratified by age of ALL in patients diagnoses between 1993 and 2012.
