# Supplementary figures and images for: Second primary acute lymphoblastic leukemia in adults: a SEER analysis of incidence and outcomes
Source: Cancer Med. 2017 Dec 28;7(2):499–507. doi: 10.1002/cam4.1266 (PMC5806098; doi:10.1002/cam4.1266)

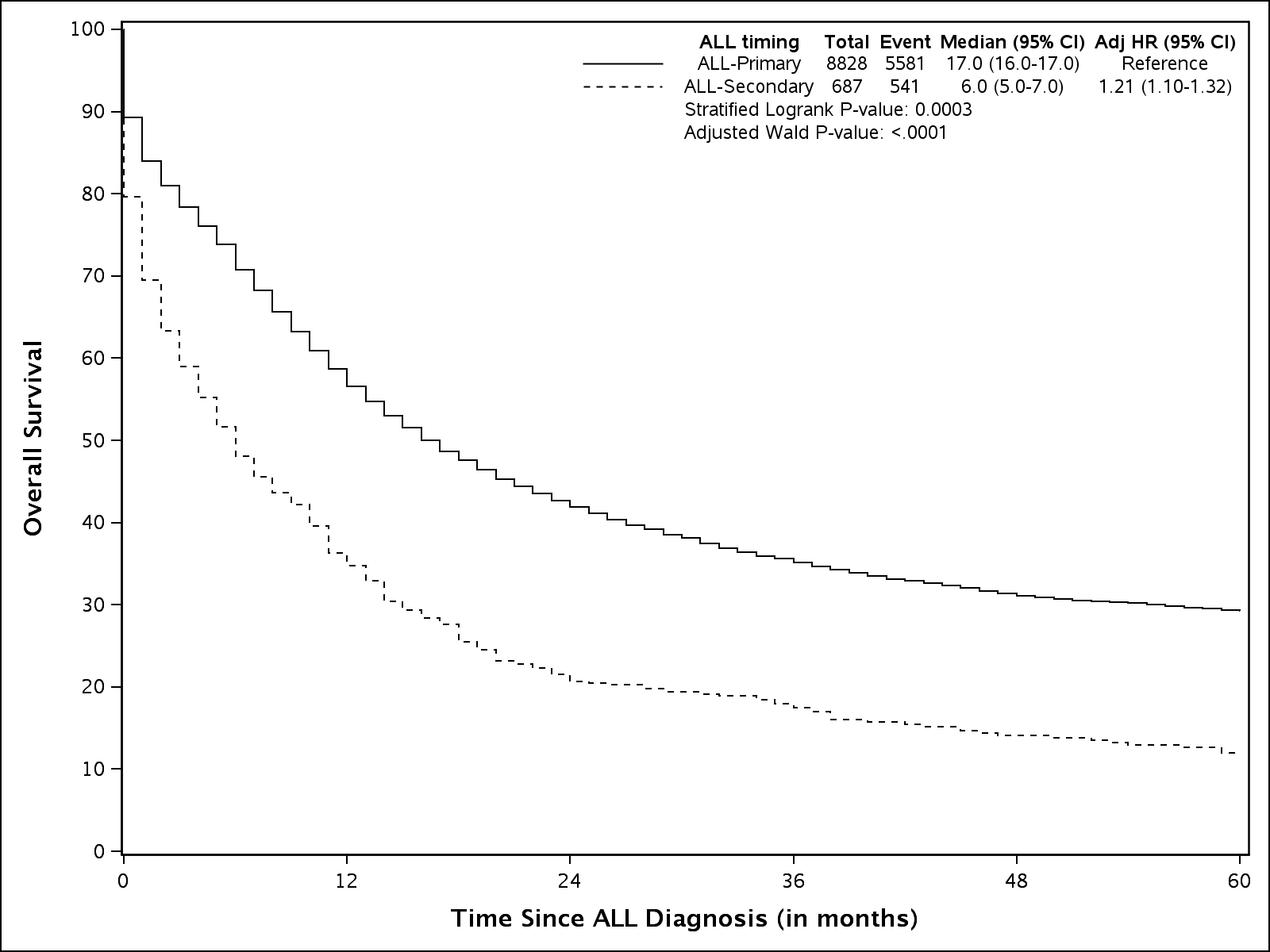

Supplement: Supplementary file 1 — Figure S1. Kaplan–Meier curves of 5‐year survival among ALL patients diagnosed between 1993 and 2012 by timing of ALL. HR is stratified on age of diagnosis and adjusted for year of diagnosis, gender, race, and SEER site. [file CAM4-7-499-s001.tif]
