# Supplementary material for: Second primary acute lymphoblastic leukemia in adults: a SEER analysis of incidence and outcomes
Source: Cancer Med. 2017 Dec 28;7(2):499–507. doi: 10.1002/cam4.1266 (PMC5806098; doi:10.1002/cam4.1266)
Supplement: Supplementary file 2 — Table S1. Demographics of 1ALL versus sALL in patients diagnoses between 1993 and 2012. Table S2. Associations with 5‐year survival using univariate and multivariate cox regression stratified by age of ALL in patients diagnoses between 1993 and 2012. [file CAM4-7-499-s002.docx]

**Supplemental Table 1. Demographics of 1ALL vs sALL In Patients Diagnoses Between 1993 and 2012**

| Characteristic | ALL de novo (N=8890) | ALL secondary (N=692) | Total (N=9582) | p-value^a^ |
| --- | --- | --- | --- | --- |
| **Gender** |  |  |  | 0.012 |
| Female | 3879 (43.6%) | 336 (48.6%) | 4215 (44.0%) |  |
| Male | 5011 (56.4%) | 356 (51.4%) | 5367 (56.0%) |  |
| **Race** |  |  |  | <0.001 |
| White | 4895 (55.1%) | 486 (70.2%) | 5381 (56.2%) |  |
| Black | 731 (8.2%) | 58 (8.4%) | 789 (8.2%) |  |
| Asian | 692 (7.8%) | 50 (7.2%) | 742 (7.7%) |  |
| Hispanic | 2441 (27.5%) | 94 (13.6%) | 2535 (26.5%) |  |
| Native American | 86 (1.0%) | 3 (0.4%) | 89 (0.9%) |  |
| Unknown | 45 (0.5%) | 1 (0.1%) | 46 (0.5%) |  |
| **Age at Diagnosis** |  |  |  | <0.001 |
| 18-44 | 4074 (45.8%) | 69 (10.0%) | 4143 (43.2%) |  |
| 45-54 | 1395 (15.7%) | 76 (11.0%) | 1471 (15.4%) |  |
| 55-64 | 1291 (14.5%) | 146 (21.1%) | 1437 (15.0%) |  |
| 65-74 | 1037 (11.7%) | 174 (25.1%) | 1211 (12.6%) |  |
| 75+ | 1093 (12.3%) | 227 (32.8%) | 1320 (13.8%) |  |
| **Year of ALL Diagnosis** |  |  |  | <0.001 |
| 1993-2002 | 2980 (33.5%) | 176 (25.4%) | 3156 (32.9%) |  |
| 2003-2012 | 5910 (66.5%) | 516 (74.6%) | 6426 (67.1%) |  |

1ALL = primary acute lymphoblastic leukemia; ALL = acute lymphoblastic leukemia; sALL = second primary acute lymphoblastic leukemia

^a^ Person Chi-square p-value

**Supplemental Table 2. Associations With Five-Year Survival Using Univariate and Multivariate Cox Regression Stratified By Age of ALL In Patients Diagnoses Between 1993 and 2012**

|  | | | **Univariate** | | **Multivariate^b^** | |
| --- | --- | --- | --- | --- | --- | --- |
| Characteristic | Total (N=9430) | Deaths (N=6065) | Hazards Ratio (95% CI) | Wald p-value^a^ | Hazards Ratio (95% CI) | Wald p-value ^a^ |
| **ALL timing** |  |  |  | 0.001 |  | <.001 |
| ALL-Primary | 8828 | 5581 (63.2%) | 1.00 (ref) |  | 1.00 (ref) |  |
| ALL-Secondary | 602 | 484 (80.4%) | 1.17 (1.07, 1.29) |  | 1.21 (1.10, 1.33) |  |
| **Year of ALL Diagnosis** |  |  |  | <.001 |  | <.001 |
| 1993-2002 | 3101 | 2324 (74.9%) | 1.00 (ref) |  | 1.00 (ref) |  |
| 2003-2012 | 6329 | 3741 (59.1%) | 0.79 (0.75, 0.84) |  | 0.78 (0.73, 0.82) |  |
| **Race** |  |  |  | <.001 |  | <.001 |
| White | 5288 | 3491 (66.0%) | 1.00 (ref) |  | 1.00 (ref) |  |
| Black | 772 | 546 (70.7%) | 1.40 (1.28, 1.53) |  | 1.46 (1.33, 1.60) |  |
| Asian | 729 | 441 (60.5%) | 1.01 (0.92, 1.12) |  | 1.04 (0.93, 1.16) |  |
| Hispanic | 2507 | 1509 (60.2%) | 1.20 (1.13, 1.28) |  | 1.19 (1.11, 1.28) |  |
| Native American | 88 | 60 (68.2%) | 1.36 (1.05, 1.76) |  | 1.26 (0.95, 1.67) |  |
| Unknown | 46 | 18 (39.1%) | 0.70 (0.44, 1.11) |  | 0.74 (0.46, 1.18) |  |
| **Gender** |  |  |  | 0.279 |  | 0.181 |
| Female | 4145 | 2728 (65.8%) | 1.00 (ref) |  | 1.00 (ref) |  |
| Male | 5285 | 3337 (63.1%) | 1.03 (0.98, 1.08) |  | 1.04 (0.98, 1.09) |  |

ALL = acute lymphoblastic leukemia; No. = number. Age of diagnosis was included as a stratification term in both univariate and multivariate models.

^a^ Overall Type-3 Wald p-value. Although the confidence intervals for some HRs may contain 1, the p-value tests to see if all levels of the covariate are equal to the referent group, or if one or more levels are significantly different.

^b^ SEER registry also included as an adjustment term in the multivariate model, data not shown
